# Survivin and Her2 Expressions in Different Grades of Urothelial Neoplasms of Urinary Bladder

**DOI:** 10.30699/IJP.2020.130859.2447

**Published:** 2020-12-21

**Authors:** Hedieh Moradi Tabriz, Elham Nazar, Seyed Ali Ahmadi, Esmaeil Azimi, Fazeleh Majidi

**Affiliations:** 1 *Department of Pathology, Sina Hospital, Tehran University of Medical Sciences, Tehran, Iran*; 2 *Research and Development Center Sina Hospital, Tehran University of Medical Sciences, Tehran, Iran *

**Keywords:** HER2, Survivin, Urothelial neoplasm

## Abstract

**Background & Objective:**

Urothelial neoplasm (UN) of bladder is a potentially lethal malignancy, particularly in locally advanced or metastatic cases. Development of molecular markers such as HER2 and Survivin may provide useful information on diagnosis and prognosis in UN of bladder.

**Methods:**

We studied the immunohistochemical (IHC) expression of HER2 and Survivin in 84 radical/partial cystectomy and transurethral resection (TUR) specimens with different histologic grades and stages. All samples were obtained from Pathology Department of Sina Hospital in Tehran, Iran from 2014 to 2018.

**Results:**

From the total number of 84 UN samples, 10 cases (11.9%) showed papillary neoplasm of low malignant potential, 30 cases (35.7%) presented with low-grade papillary urothelial neoplasm, and 44 cases (52.4%) diagnosed as high-grade papillary urothelial neoplasm. HER2 and Survivin expressions were seen in 44 (52.4%) (*P*=0.610) and 9 (10.7%) patients (*P*=0.046), respectively. Survivin expression showed a mild increase in high grade UN.

**Conclusion:**

Our findings suggest that the IHC expression of Survivin and HER2 are not well associated with histological grades of urothelial neoplasms of bladder. This may be partly due to relatively small sample size and other factors such as patient characteristics or antibody specifications.

## Introduction

Urothelial neoplasm (UN) is the 9th most common cancer and one of the important leading causes of cancer-related death worldwide (1, 2). It is identified as the fourth and eleventh most common type of malignancy in males and females, respectively (3). UC risk factors are well known (4). At one end of the range, there are low-grade tumors which have a low developing rate and need preliminary endoscopic treatment and observation but rarely present a warning to the patient. At the other extreme, there are high-grade tumors which have a high malignancy potential associated with considerable development and high cancer mortality rates (5). Also 80% of UN cases are diagnosed as non-muscle invasive bladder cancer (NMIBC), in which up to 50% of the cases would experience recurrence and 20% would progress within 5 years (6, 7). So, UNs are categorized based on histopathologic grading and muscle invasion. The majority of them are non-invasive low-grade tumors (1). UN management is mostly based on grading and staging of the tumor, but they are not sufficient to predict the patients’ outcome (8). Standard methods are available for UN diagnosis and prognosis assessment (8). These methods are expensive and uncomfortable, so biochemical markers as alternative diagnostic methods, have been developed (9, 10). Some biochemical markers are considered to be more sensitive and specific in the target population compared with routine examination for the prognosis assessment. Also, evaluation of biochemical markers is safe, non-invasive, and easy to use; however, it is not cheap and available (8, 10). Some studies have suggested that Survivin is an apoptosis’ inhibitor protein which blocks caspase activation (6, 11, 12). In cancer cells, Survivin is expressed in the G2/M phase of the cell cycle and counteracts apoptosis induction during mitosis by interfering with the function of caspases (11). Survivin is over expressed in several human malignancies leading to tumor progression and metastasis. Likewise, in UN, Survivin expressed in tumoral tissue causes rapid progression of the disease and increased rates of recurrence (13). So Survivin, an apoptosis inhibitor protein, is a useful marker for prognostic assessments in invasive bladder tumors (14). Functionally, Survivin inhibits apoptosis, promotes cell proliferation, and induces/enhances angiogenesis. In transitional cell carcinoma of the urinary bladder, Survivin has been shown to be a promising biomarker for cancer diagnosis, prognosis, and prediction of possible response to intravesical or systemic therapies (15). Because of its expression in cancer but not in normal tissues, we investigated the potential suitability of Survivin immunostaining as a new molecular marker for prognosis evaluation of UN (16). Human epidermal growth factor receptor-2 (HER2) is a member of tyrosine kinase receptor family. The epidermal growth factor (EGF) family of receptor tyrosine kinases comprises four members: HER1 (EGF receptor 1 Human EGF Receptor/ErbB1), HER2 (neu/ErbB2), HER3 (ErbB3) and HER4 (ErbB4). EGF receptors are commonly active in a dimeric form and interaction between different EGF receptor pairs represents a signal diversification and amplification mechanism (1). Although it is low expressed in normal tissues, it has a significant role in the pathogenesis of some cancers (17). HER2 over-expression particularly characterizes aggressive cancer types of various origins with poor outcome (18). Only a few studies have been conducted regarding the importance of HER2 status in UN, so there is still controversy over this subject (19). Our study aims to evaluate HER2 and Survivin expressions using immunohistochemical (IHC) analysis and their association with histologic grade of UNs.

## Materials and Methods

**Sample Selection**

A total number of 84 cases of transitional bladder tumor were selected from the pathology archive of Sina Hospital affiliated to Tehran University of Medical Sciences from 2014 to 2018. The specimens were obtained via transurethral resection (TUR) and cystectomy (radical or partial). Patients’ records were also considered for demographic data. This study was cross sectional. Cases with incomplete information were excluded. Slides were re-assessed by an expert pathologist who was unaware of the patients’ history. After confirmation of diagnosis, appropriate blocks of each sample were selected. The specimens were classified according to the grade of tumor progression elaborated by World Health Organization (WHO)/International Society of Urological Pathology (ISUP) criteria approved in 2016 (20). Tumor staging was also performed according to the College of American Pathologists (CAP) guideline. Other assessed items included muscle invasion, perineural invasion, lymphatic invasion, blood vascular invasion, multifocality or unifocality of tumor and presence of in situ component (21). 

**Immunohistochemistry Staining**

**Fig. 1:**
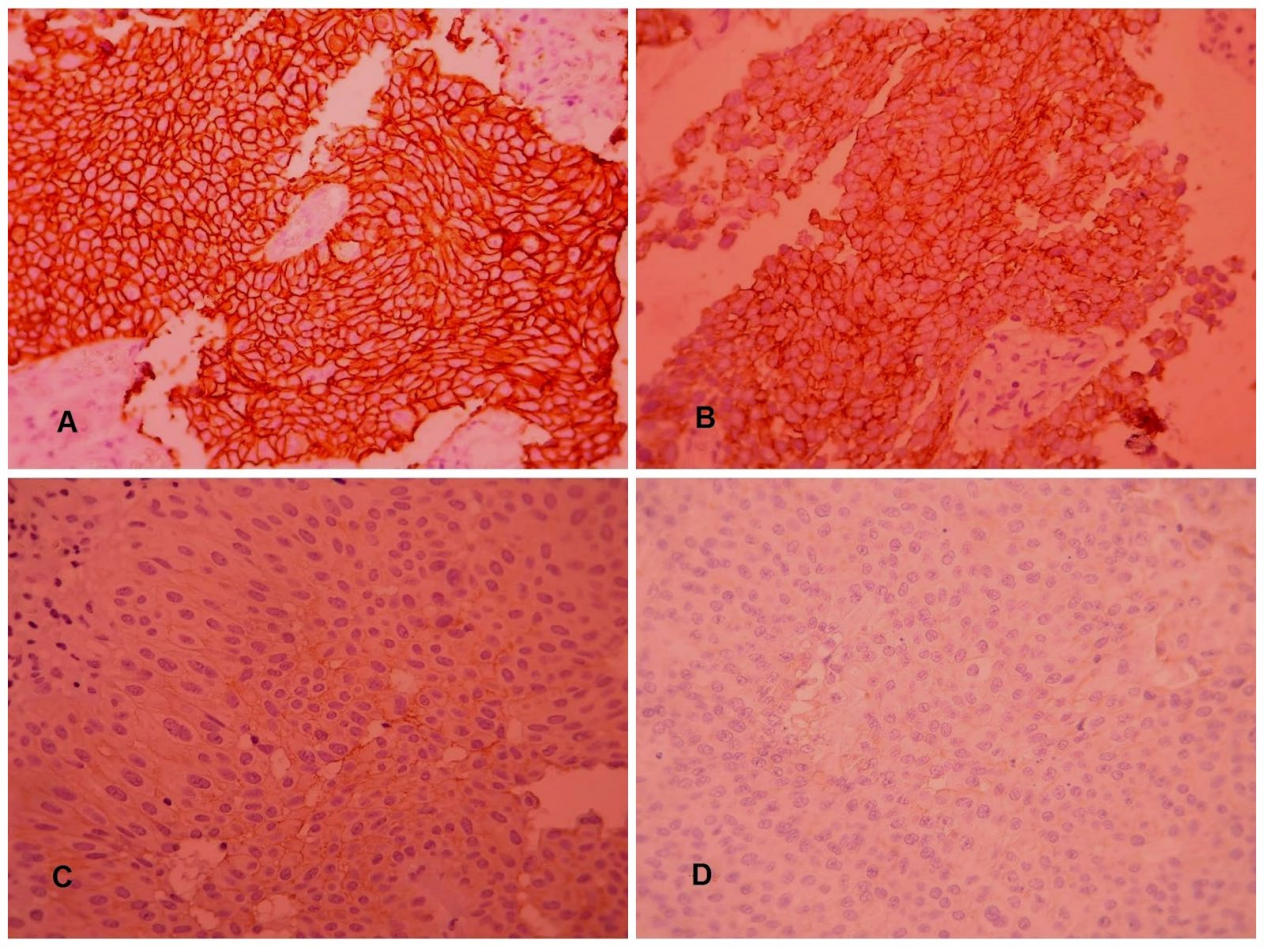
Her2/neu IHC stain scoring in tumoral cells according to the Table 1: (A) score +3, (B) score +2, (C) score +1, (D) score 0

**Fig. 2:**
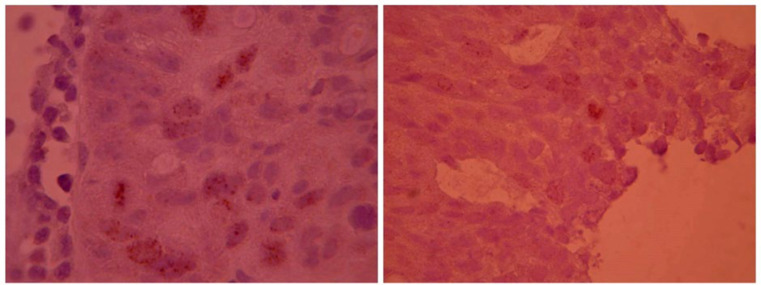
Positive Survivin IHC staining in 10% of nuclear tumoral cells

We prepared 2 mm thick paraffin blocks. Slides were coated with Poly-L-lysine and then deparaffined and rehydrated. IHC staining was performed on one representative slide per tumor which exhibited a maximum of tumoral tissue in order to detect HER2 and Survivin expression. A 1:250 dilution of primary antibody (Polyclonal rabbit anti-human C-erb B-2, Dako) was prepared according to the manufacturer’s protocol for HER2 staining. A 1:100 dilution of Survivin (clone12c4, Dako, USA-monoclonal mouse anti human) was also prepared according to the manufacturer’s protocol. Hematoxylin was used to stain the background. Breast carcinoma samples positive control was used to evaluate HER2. HER2 expression staining was graded according to Table 1 (22) (Figure 1). For Survivin, tumors which showed increased expression in more than 10% of the nuclear tumoral cells, were considered positive in our study (23, 24) (Figure 2).

**Statistical Analysis**

Statistical analysis of the results was performed using SPSS-18 software (SPSS Inc., Chicago, IL., USA). The HER2 and Survivin expressions were evaluated in general as well as correlation with tumor grade of the disease and other variables including age, sex, and muscular layer invasion. T-test and Chi-square tests were used for data analysis. All statistical tests were two-sided, and P-value<0.05 was considered significant.

## Results

The study included 84 cases comprised of 76 males (90.5%) and 8 females (9.5%) with the average age of 62.6 years and standard deviation of 13.1 years (33-99 years). 30 cases (35.7%) underwent radical cystectomy, 5 cases (5.9%) underwent partial cystectomy, and 49 cases (58.3%) underwent transurethral resection of bladder tumors (TURBT).

Out of 84 cases, 10 cases (11.9%) showed papillary neoplasm of low malignant potential, 30 cases (35.7%) presented with low-grade papillary urothelial carcinoma, and 44 cases (52.4%) were diagnosed as high-grade papillary urothelial carcinoma. Our study included all grades of the cancer (low to high). Distribution of the cases with urothelial neoplasms according to the T-stage consists of 39 (46.4%) cases with muscle invasion (stage T2a, T2b, T3, T4a) and 45 cases (53.6%) without muscle invasion (stage Ta, T1). 

Out of 84 cases with bladder papillary neoplasm, 44 cases (52.4%) (*P*=0.610) showed HER2 expression and 9 (10.7%) (*P*=0.046) cases showed Survivin expression (Table 2). HER2 expression staining degree ranges from 0 to +3 according to Table 1.

**Table 1:** HER2 expression staining grade according to the positive control of breast carcinoma samples (21).

Staining pattern	score	HER2 overexpression assessment
No staining is observed, or membrane staining is observed in less than 10% of tumor	0	Negative
A faint/barely perceptible membrane staining is detected in more than 10% of tumor cells. The cells are only stained in part of their membrane	1+	Negative
A weak to moderate complete membrane staining is observed in more than 10% of the tumor cells	2+	Weakly positive
A strong complete membrane staining is observed in m than 10% of tumor	3+	Strongly positive

**Table 2:** Demographic distribution of the cases

Total case	Frequency	percent
**Sex**		
Male	76	90.5%
Female	8	9.5%
**Resection type**		
Radical cystectomy	30	35.7%
Partial cystectomy	5	5.9%
TUR	49	58.3%
**Carcinoma type**		
papillary neoplasm of low- malignant potential	10	11.9%
low-grade papillary urothelial carcinoma	30	35.7%
high-grade papillary urothelial carcinoma	44	52.4%
**T-stage**		
Ta	16	19.0%
T1	29	34.5%
T2a	18	21.4%
T2b	8	9.5%
T3	6	7.1%
T4a	7	8.3%
**Muscle invasion**		
+	39	46.4%
-	45	53.6%
Perineural invasion	13	45.5%
lymphatic invasion	21	25%
blood vessels invasion	13	45.5%
multi-centric tumor	18	21.4%
Insitu component	4	4.8%
**Markers**		
HER2	44	52.4%
Survivin	9	10.7%

The frequency of HER2 expression in different grades of urothelial neoplasms including papillary neoplasm of low malignant potential, low-grade papillary urothelial carcinoma and high-grade papillary urothelial carcinoma were 40%, 50%, 56.8%, respectively (Table 3), which were not statistically significant (*P*=0.597) by Chi-square analysis.

The frequency of Survivin in different grades of urothelial neoplasms including papillary neoplasm of low malignant potential, low-grade papillary urothelial carcinoma and high-grade papillary urothelial carcinoma was 10%, 0%, and 15.9%, respectively (Table 3), which indicates a mild increasing trend in Survivin expression in high grade UN (*P*=0.057) by Chi-square analysis.

The frequency of HER2 expression was 48.7% in urothelial neoplasms which showed invasion into the muscle layers and 55.6% in non-invasive cases (Table 3), which was not statistically significant (*P*=0.531) by Chi-square analysis.

Survivin expression was 12.8% in urothelial neoplasms with muscle layers invasion and 8.9% in non-invasive cases (Table 3), which was not statistically significant (*P*=0.561) by Chi-square analysis.

The frequency of HER2 and Survivin was evaluated considering variable factors such as patients’ sex, age, tumor size, perineural invasion, vascular invasion and lymph node involvement, tumor multicentricity, and presence of in situ regions. There was no statistically significant relation between the frequency of these markers and the above variables (Table 3) using T-test analysis (*P*>0.500). .

**Table 3:** Positive and negative HER2 and Survivin markers frequency rates

	HER2 positive	HER2 Negative	P-value	Survivin Positive	Survivin Negative	P-value
Immunohistochemistry staining			0.0610			
0	20 (23.8%)					
1	20 (23.8%)					
2	41 (48.8%)					
3	3 (3.6%)					
Different tumor grades			0.269			0.046
Papillary neoplasm of low- malignant potential	4 (40%)	6 (60%)		2 (10%)	8 (80%)	
Low-grade papillary urothelial carcinoma	15 (50%)	15 (50%)		0	30 (100%)	
High-grade papillary urothelial carcinoma	25 (56.8%)	19 (43.3%)		7 (15.9%)	37 (84.1%)	
Gender			0.662			0.590
Male	38 (50%)	38 (50%)		9 (11.8%)	67 (88.2%)	
Female	6 (75%)	2 (25%)		0	8 (100%)	
Muscle invasion			0.662			0.727
+	19 (48.7%)	20 (51.3%)		5 (12.8%)	34 (87.2%)	
-	25 (55.6%)	20 (44.4%)		4 (8.9%)	41 (91.1%)	
Perineural invasion			0.765			0.100
+	6 (46.2%)	7 (53.8%)		1 (7.7%)	12 (92.3%)	
-	38 (53.5%)	33 (46.5%)		8 (11.3%)	63 (88.7%)	
lymphatic invasion			0.801			0.100
+	10 (47.6%)	11 (52.4%)		2 (9.5%)	19 (90.5%)	
-	34 (54%)	29 (46%)		7 (11.1%)	56 (88.9%)	
blood vessel invasion			0.765			0.100
+	6 (46.2%)	7 (53.8%)		1 (7.7%)	12 (92.3%)	
-	38 (53.5%)	33 (46.5%)		8 (11.3%)	63 (88.7%)	
Multicentricity			0.109			0.100
+	6 (33.3%)	12 (66.7%)		2 (1.1%)	16 (88.9%)	
-	38 (57.6%)	28(42.4%)		7 (10.6%)	59 (89.4%)	
In-situ component			0.343			0.370
+	1 (25%)	3 (75%)		1 (25%)	3 (75%)	
-	43 (53.1%)	37 (46.3%)		8 (10%)	72 (90%)	

## Discussion

The role of HER2 in bladder cancer remains controversial (16). Increased expression of the EGF receptors, and HER2 is related to poor prognosis in most cancer studies (25). Recently, with the advent of recombinant humanized monoclonal anti-HER2 antibody (trastuzumab, Herceptin), assessment of HER2 expression has gained therapeutic significance (17). At present, targeted anti-HER2 therapies are established clinical routines for HER2 over-expressing/amplified carcinomas of the breast and stomach. Recent studies have evaluated HER2 status in urothelial neoplasms to assess the therapeutic potential of this target, demonstrate significant protein over-expression (score 2+ or 3+) or gene amplification in approximately 10 % of the tumors (26). Expression of Survivin, an apoptosis inhibitor protein, is up-regulated in many tumors of epithelial origin and frequently associates with disease prognosis (27). Detection of Survivin or its associated gene signature may provide an early biomarker of aggressive tumor behavior before the appearance of tissue abnormalities (28). Several studies on UN have indicated that there may be a relationship between Survivin expression and ultimate behavior of the carcinoma, although the exact nature of this relationship is still not fully understood, because the results of some of these studies seem to be contradictory (29). There are a few conflicting reports regarding the association of Survivin expression with recurrence rates in non-muscle invasive bladder cancers (30). Also, Survivin proteins were identified as strong independent prognostic factors in patients with advanced bladder cancer (31). According to some related research, HER2 expression in metastasizing urothelial neoplasm is relatively frequent, homogeneous in each tumor component, and predicts poor prognosis (32). But some studies suggest that analyzing the HER2 status does not indicate any prognostic information in patients with UN (27). Therefore, we determined the association of Survivin and HER2 expression with clinical and pathologic characteristics in patients with different grades in UN. Our study showed high incidence rate of Survivin expression in high-grade papillary urothelial carcinoma. This result was similar to some recent studies (14, 31, 33, 34). There was no significant relation between HER2 incidence and grade of UN. For HER2 evaluation, Fluorescence in situ hybridization (FISH) might be more accurate than IHC staining and this is a possible cause of different results in some studies compared to our study (21).

## Conclusion

Survivin and HER2 expressions evaluation in different histopathologic grades of urothelial neoplasms of urinary bladder suggest that the IHC expression of Survivin and HER2 are not associated with histological grade of urothelial neoplasms of bladder. This may be in part due to the relatively small sample size and other factors such as patient characteristics or antibody specifications.
